# Meckel-Gruber syndrome: a rare and fatal congenital disorder (case report)

**DOI:** 10.11604/pamj.2025.52.162.47620

**Published:** 2025-12-16

**Authors:** Khouloud Moulehi, Imen Bannour, Chayma Rjiba, Salma Ben Youssef, Manel Kaabi, Mohamed Mallouli, Hafedh Touileb, Mohamed Mnasri, Sassi Bouguizéne, Badra Bannour

**Affiliations:** 1 Department of Gynecology and Obstetrics, Farhat Hached University Hospital, Sousse, Tunisia,; 2 Faculty of Medicine, Ibn El Jazzar University of Sousse, Sousse, Tunisia

**Keywords:** Meckel-Gruber syndrome, renal dysplasia, oligohydramnios, prenatal diagnosis, case report

## Abstract

Meckel-Gruber syndrome is a rare congenital disorder characterized by multiple malformations. It transmits via a recessive autosomal mode. It is characterized by an occipital encephalocele, polydactyly, and polycystic renal dysplasia. The diagnosis could be established via ultrasound. In fact, it is actually the key method for the early screening of this lethal malformation with at least two of its main characteristics. However, the diagnosis is confirmed by karyotype analysis. Herein, we present a case of Meckel-Gruber syndrome diagnosed in a fetus from a consanguineous marriage in a 40-year-old woman, gravida 2 para 1, with one previous healthy child. This pregnancy was terminated at 19 weeks of gestation. The diagnosis was made through prenatal ultrasound and magnetic resonance imaging (MRI). With later confirmation by fetal autopsy.

## Introduction

Meckel-Gruber syndrome was first reported by Meckel in 1822, with further characterization provided by Gruber in 1934. It is a recessive autosomal fatal condition [1]. The classic triad encompasses polycystic kidneys, occipital encephalocele, and polydactyly [2]. Prenatal screening is usually done via ultrasound between the 10^th^ and 14^th^ weeks of gestation. The detection of this alteration is crucial to diagnose and propose genetic counseling [1]. Thus, a well-managed pregnancy termination should be the standard in case of lethal malformations [3].

## Patient and observation

**Figure 1:**
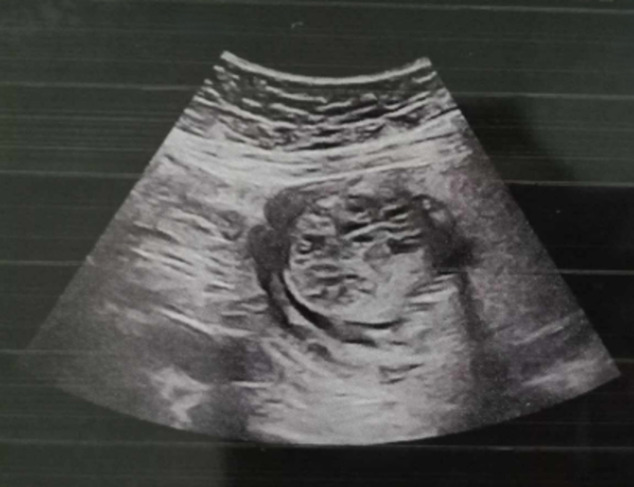
a prenatal ultrasound showing symmetrical bilateral nephromegaly with poly-cystic renal dysplasia

**Patient information:** a 40-year-old female patient, gravida 2, para 1, with one previous cesarean delivery of a healthy living child, was referred to our hospital by another medical facility at 19 weeks of gestation due to the ultrasonographic detection of an abnormal bilateral and symmetric nephromegaly with multi-cystic renal dysplasia associated with occipital meningocele (Figure 1). These observed lesions were characteristic features pointing to Meckel-Gruber syndrome.

**Clinical findings:** the patient was conscious and hemodynamically stable. The obstetrical examination was normal.

**Timeline of current episode:** the clinical course unfolded rapidly, with diagnosis and intervention completed within a short gestational window.

**Figure 2:**
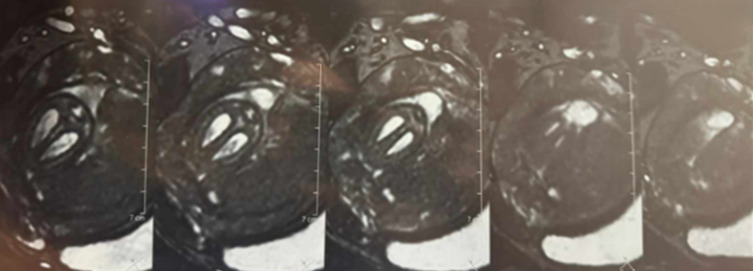
fetal MRI showing an occipital sub-torcular meningocele

**Figure 3:**
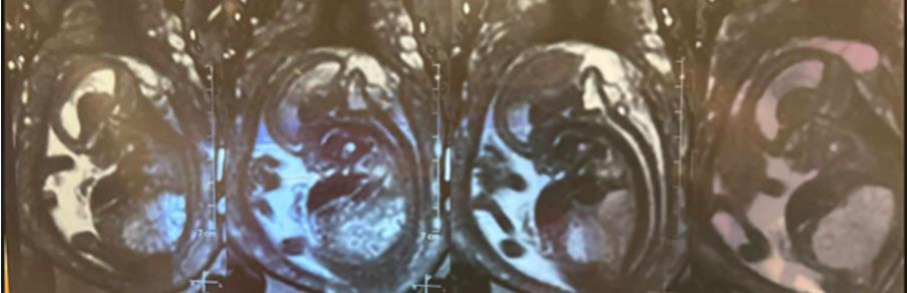
bilateral nephromegaly (heterogenous T2 hyperintensity) measuring 40 x 20mm on the right side and 35 x 18mm on the left side

**Diagnostic assessment:** a fetal MRI was performed for a better description of fetal lesions. It showed a subcutaneous occipital liquid formation communicating with the posterior fossa through an opening measuring 23 x 10mm, in line with a sub-torcular meningocele (Figure 2). It also highlighted the presence of a heterogeneous T2 hyperintense bilateral nephromegaly measuring 40 x 20mm on the right side and 35 x 18mm on the left side (Figure 3). The bladder was not visualized, and there was severe oligohydramnios.

**Diagnosis:** given the lethal outcome of this syndrome, the therapeutic abortion of the pregnancy was decided by a multidisciplinary team meeting and was approved by the couple.

**Therapeutic interventions:** the patient was administered misoprostol at a dosage of 400 µg every 6 hours.

**Figure 4:**
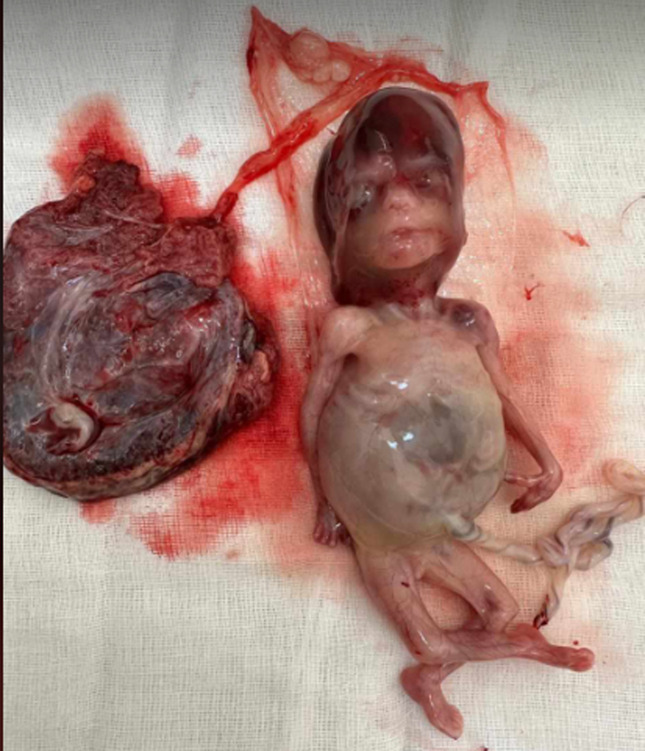
fetus exhibiting multiple malformations consistent with Meckel-Gruber syndrome

**Figure 5:**
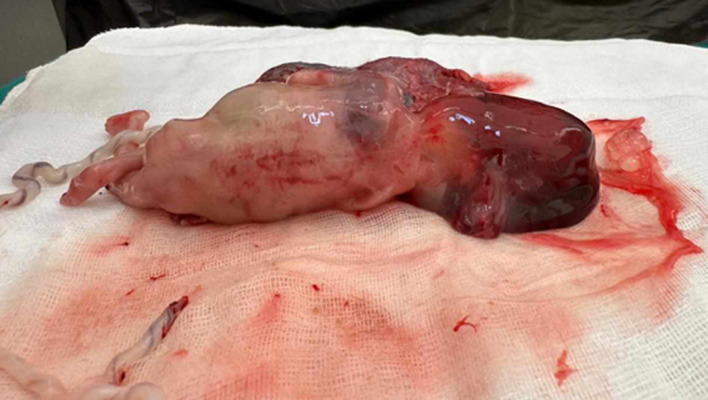
a posterior fetal view showing occipital encephalocele

**Figure 6:**
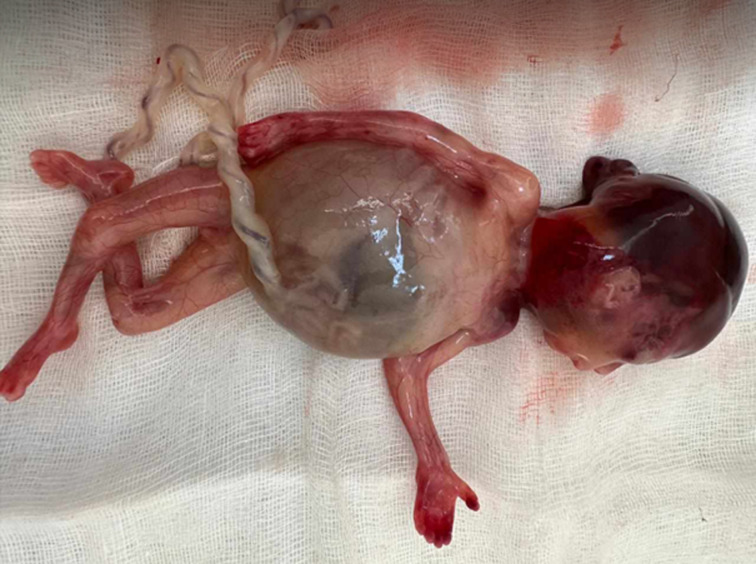
abdominal distension with bilateral nephromegaly

**Follow-up and outcome of interventions:** after fetal expulsion, the gross examination showed a female fetus weighing 120g (Figure 4) and exhibiting an occipital encephalocele (Figure 5). An abdominal distension along with bilaterally palpable kidneys (Figure 6). Polydactyly, cleft lip or palate, and genital malformations were not noted.

**Patient perspective:** *“Hearing that my baby had a fatal condition was devastating. I had no complications before, so the diagnosis came as a shock. Choosing to end the pregnancy was incredibly difficult, but the medical team supported us with compassion and clarity. I´m grateful for their kindness during such a painful time. Though I still carry the grief, I take comfort in knowing our experience may help others through early diagnosis and awareness”*.

**Informed consent:** informed consent was obtained from the patient for the fetal autopsy procedure and for the histopathological examination.

## Discussion

Meckel-Gruber syndrome is a recessive autosomal fatal illness. It usually manifests in a triad including bilateral cystic kidneys, central nervous system malformations, and polydactyly [4].

Meckel-Gruber syndrome has an estimated incidence ranging from 1 in 13,250 to 1 in 1,140,000 individuals worldwide. To date, three genes have been implicated in its pathogenesis: MKS1, located on chromosome 17, MKS2, on chromosome 11, and MKS3, on chromosome 8 [5].

**Clinical manifestation:** Meckel syndrome is a hereditary malformation syndrome resulting from a monogenic defect, typically combining renal cystic disease with multiple additional congenital anomalies [5]. Meckel syndrome is classically defined by a diagnostic triad consisting of occipital encephalocele, renal cystic dysplasia, and polydactyly [6].

The polydactyly is most frequently post-axial, characterized by the presence of an additional digit on the ulnar or fibular side (commonly a sixth finger), although in some cases it may present as pre-axial, such as duplication of the thumb [7]. In approximately one out of six cases, a curvature or bowing of the long bones of the limbs can also be observed. In addition to these core features, a variety of other congenital anomalies have been reported, including cleft lip and/or palate, ocular abnormalities such as anophthalmia or microphthalmia, urethral atresia, congenital heart defects, and malformations of the external and internal genital organs [8].

**Diagnostic criteria:** the diagnosis is based on both major and minor criteria. The major criterion includes cystic renal dysplasia, which is essential for confirming the diagnosis. This renal abnormality is characterized by the presence of cysts of varying sizes, often associated with fibrosis of the renal parenchyma, hypoplasia, or aplasia of the renal units. Cystic renal dysplasia may be unilateral or bilateral, with the bilateral form often leading to severe renal insufficiency [8].

Oligohydramnios is frequently observed and is associated with this condition. It can serve as an indirect sign of impaired renal function, typically due to a reduction in fetal urine output. The decrease in amniotic fluid volume is commonly linked to renal anomalies, including cystic renal dysplasia, and may contribute to the early recognition of the disorder during routine prenatal assessments [5]. Oligohydramnios may also be associated with significant fetal complications, including pulmonary hypoplasia, due to the reduced volume of amniotic fluid necessary for normal lung development [6].

Minor criteria may include renal structural anomalies, such as renal hypoplasia or abnormal ureteral morphology, although these findings alone are insufficient for a definitive diagnosis. The diagnosis is typically confirmed through a combination of these clinical and imaging criteria, with prenatal ultrasound being the primary modality, supplemented by fetal MRI in certain cases for further clarification [7]. On the other hand, minor criteria encompass liver fibrosis, occipital encephalocele, polydactyly, and other central nervous system malformations like Dandy-Walker and Arnold-Chiari malformations [3].

**Prenatal diagnosis:** prenatal screening can be performed using ultrasonographic imaging, which may reveal an intracranial anechoic cyst and/or the identification of cranial malformations at the end of the first trimester. Additionally, the presence of abnormally large kidneys can be detected, which further aids in the early diagnosis of the condition. These findings, when observed together, provide crucial indicators that warrant closer monitoring and confirmatory diagnostic procedures [2].

Other features of the syndrome may become apparent in subsequent ultrasound examinations, often detected at later stages of pregnancy. These characteristics may include additional structural anomalies or abnormalities in fetal development, which can help refine the diagnosis and guide clinical management [3]. Karyotyping remains the most efficient diagnostic tool, serving as the cornerstone for its confirmation [1].

**Differential diagnosis:** multiple malformative disorders may cause more serious diagnostic challenges. The Carpenter-Hunter syndrome also includes encephalocele, cystic renal dysplasia, and polydactyly. However, it also shows generalized bone lesions [7]. A huge help in the diagnosis of this fetal disorder is provided by the isolation of the gene for Meckel syndrome [6].

**Genetic counseling:** Meckel-Gruber syndrome is transmitted through a recessive autosomal pattern. Genetic counseling´s main purpose is to inform parents experiencing such a diagnosis for their infant that the risk of recurrence is 25% for the next pregnancy. The frequency of the Meckel gene is estimated at 1/400 in the general population [8].

## Conclusion

Meckel-Gruber syndrome is a rare but devastating genetic alteration, characterized by a classic triad including encephalocele, multicystic renal dysplasia, and polydactyly. Progress in understanding the genetic foundations and pathophysiological mechanisms has improved our ability to diagnose and counsel affected families. Ongoing continuous research is mandatory for the development of potential therapeutic approaches and enhancing the quality of life for the families.

## References

[1] Al-Belushi M, Al Ibrahim A, Ahmed M, Ahmed B, Khenyab N, Konje JC (2016). A review of Meckel-Gruber syndrome-incidence and outcome in the state of Qatar. J Matern Fetal Neonatal Med.

[2] Vernekar JA, Mishra GK, Pinto RG, Bhandari M, Mishra M (1991). Antenatal ultrasonic diagnosis of Meckel Gruber syndrome (a case report with review of literature). Australas Radiol.

[3] Audifred-Salomón J, Barrita-Domínguez IJ, Ortiz de ZA, Sánchez-Hernández H, Camacho-Cervantes A (2016). Prenatal diagnosis of Meckel-Gruber syndrome. Case report and literature review. Ginecol Obstet Mex.

[4] Patil PR, Polisgowdar AB (2023). EP11. 18: Early diagnosis of Meckel–Gruber syndrome on transabdominal ultrasound: a case report. Ultrasound in Obstetrics & Gynecology.

[5] Shireesha C, Manasa A (2022). Meckel Gruber Syndrome-A Rare Case Report.

[6] Walsh M, Graupman P (2006). Meckel-Gruber syndrome in association with an occipital meningocele. Pediatr Neurosurg.

[7] Jondhale P, Marda M, Bangal VB, Bagdi N (2020). Meckel-Gruber syndrome: a rare and lethal foetal anomaly. Int J Reprod Contracept Obstet Gynecol.

[8] Wade M, Gueye M, Mbodji A, Ndiaye MD, Sene M, Mbaye M (2022). Meckel-Gruber syndrome: about a case identified during deliver. International Journal of Reproduction, Contraception, Obstetrics and Gynecology.

